# Plants Grown in Parafilm-Wrapped Petri Dishes Are Stressed and Possess Altered Gene Expression Profile

**DOI:** 10.3389/fpls.2019.00637

**Published:** 2019-05-15

**Authors:** Le Xu, Shengjie Li, Sergey Shabala, Tao Jian, Wenying Zhang

**Affiliations:** ^1^Hubei Collaborative Innovation Centre for Grain Industry/Engineering Research Centre of Ecology and Agricultural Use of Wetland, Ministry of Education, Yangtze University, Jingzhou, China; ^2^Guangdong Provincial Key Laboratory of Biotechnology for Plant Development, School of Life Sciences, South China Normal University, Guangzhou, China; ^3^Tasmanian Institute for Agriculture, University of Tasmania, Hobart, TAS, Australia

**Keywords:** *Arabidopsis*, transcriptome analysis, gas exchange, abiotic stress, sterile culture

## Abstract

*Arabidopsis* is used as a model species in numerous physiological and genetic studies. Most of them employ parafilm-wrapped sterile culture. Here we demonstrate that this method is prone to potential artifacts and can lead to erroneous conclusions. We compared the effect of different sealing methods including air-permeable paper tape and traditional parafilm on *Arabidopsis* seedling growth, root development and gene expression network. Although seedlings grown in Petri dishes after 1 week sealed with paper tape showed a similar growth phenotype to that of parafilm-sealed seedlings, more than 700 differentially expressed genes (DEG) were found, including stress and nutrition-responsive genes. In addition, more H_2_O_2_ was accumulated in the tissues of parafilm-sealed plants. After 14 days of growth, paper tape-sealed plants grew much better than parafilm-sealed ones and accumulated higher chlorophyll content, with 490 DEGs found. After 3 weeks of growth, paper tape-sealed plants had higher chlorophyll and better growth compared to parafilm-sealed ones; and only 10 DEGs were found at this stage. Thus, the obvious phenotype observed at the latter stage was a result of differential gene expression at earlier time points, mostly of defense, abiotic stress, nutrition, and phytohormone-responsive genes. More O_2_ content was detected inside paper tape-sealed Petri dishes at early growth stage (7 days), and distinct difference in the CO_2_ content was observed between parafilm-sealed and paper tape-sealed Petri dishes. Furthermore, the carbon source also influenced seedlings growth with different sealing methods. In conclusion, conventional sealing using parafilm was not the optimal choice, most likely because of the limited gas exchange and a consequent stress caused to plants.

## Introduction

*Arabidopsis thaliana* belongs to the Cruciferae and is known as a “plant fly” due to its broad use in genetic studies and the fast growth cycle, simple morphological characteristics, as well as strong vitality. The genome sequence of *Arabidopsis* *thaliana* was the first amongst plants to be completely sequenced and published at the end of 2000. *Arabidopsis* also has a large mutant library and has long been used as a model organism in biological studies. The results of *Arabidopsis* *thaliana* research in botany can help deepen the understanding of other plants and improve the application of *Arabidopsis* to important crops (Meinke et al., 1998; Eckardt, 2001).

Plant tissue culture is a tool for basic research and with potential applications in agriculture, horticulture, and forestry industry of important plant bioactive compounds. Plant tissue culture has wide application, encompassing vector and vector-free genetic transformation of cells, embryo rescue, somatic embryogenesis, and commercial clonal propagation. Much plant biotechnology requires at least one stage where plant tissue culture is obligatory. Because of the short cycle and sensitivity to various stimuli, many researchers choose sterile culture to study seed germination rates and response to phytohormones or other chemical substances (Thorpe, 2006), as well as use Petri dish-grown plants to study mechanisms of tolerance to a broad range of abiotic stresses such as salinity or heavy metal toxicity.

The requirement for both sterility and the avoidance of dehydration in plant tissue cultures can impose sealing requirements that severely limit the rate of gas exchange in and out of the culture vessel. Sealing with parafilm to protect tissue cultures from infection and desiccation is the most used method. However, the use of the parafilm may potentially interfere with the free exchange of gasses between the cultured material and the outside atmosphere. This shortcoming can have unwelcome consequences for culture performance because of the strong physiological impact of the gasses involved, notably, O_2_, CO_2_, and C_2_H_4_ (Jackson et al., 1994). Oxygen and CO_2_ are principal substrates or products of aerobic respiration and photosynthesis and thus intrinsic to the most basic life-sustaining metabolic pathways of plant cells.

Despite the self-evident importance, researchers often neglect the gaseous environment for tissue cultures and used parafilm-sealed Petri dishes, for convenience purpose. Here we show that this approach is flawed, as it may result in an altered phenotype, gas composition, and gene expression pattern, hence, make a major impact on data interpretation.

## Materials and Methods

All wild-type (ecotype Col-0) were surface sterilized with 20% bleach containing 0.1% Tween 20 for 15 min and then washed approximately five times with sterilized water. Seeds were sown on Murashige and Skoog medium, followed by cold treatment in the dark for 2 days. For sucrose deficiency treatment, all the preparations for the MS medium were the same except no addition of sucrose. MS medium composition was as follows: potassium nitrate, 1.9 g; ammonium nitrate, 1.65 g; calcium chloride, 0.33 g; potassium phosphate monobasic, 0.17 g; magnesium sulfate, 0.18 g; boric acid, 0.0062 g; manganese sulfate monohydrate, 0.0169 g; molybdic acid (sodium salt), 0.00025 g; zinc sulfate heptahydrate, 0.0086 g; potassium iodide, 0.00083 g; copper sulfate pentahydrate: 0.000025 g; ferrous sulfate heptahydrate, 0.0152 g; myo-inositol, 0.1 g; glycine, 0.002 g; thiamine hydrochloride, 0.0001 g; pyridoxine HCl, 0.005 g; niacin, 0.0005 g; ethylene diamine tetraacetic acid, 0.03726 g. MS powder was purchased from Solarbio (Beijing, Cat NO. M8521). For 1 L MS medium, 4.42 g powder was used and 8 g agarose and 20 g sucrose were added. Media pH was adjusted to 6.5–6.6 with 1N NaOH/HCl (not buffered). Parafilm was purchased from Parafilm M Laboratory Film, and the paper tape was from 3M Yuwell company (China). For the parafilm we used 10.2 cm parafilm in length, and we wrapped the Petri dish around the circumference twice. The same was done for the paper tape. The single-use plastic Petri dishes were used. Light intensity was 150 umol s^-1^ m^-2^. Around thirty uniform seedlings were grown on each Petri dishes. Seedlings were grown in a plant growth room under a 16-h light/8-h dark cycle at 22°C.

### Gas Content, Water Content, and pH Value Measurement

Gas detector tubes (GASTEC, Japan) were used to measure the gas content in the Petri dish with seedlings after growing for 1, 2, and 3 weeks. The detecting layer of the tubes contained reaction particles and chemical reactions happen when they meet certain type of gas. For oxygen, measuring range was 6–24%, the color of detecting layer changed from black to white and the reaction principle is O_2_+4TiCl_3_+6H_2_O→4TiO_2_+12HCl.

For CO_2_, the measuring range is 100–2000 ppm and the color of detecting layer changed from pale red to orange, as a result of the following chemical reaction: CO_2_+2KOH→K_2_CO_3_+H_2_O. For ethylene, the measuring range was 0.2–50 ppm and the color of detecting layer changed from pale yellow to blue following CH_2_:CH_2_+PbSO_4_+(NH_4_)_2_MoO_4_→Molybdenum blue reaction. The Gastec sampling pump together with Gastec detector tubes were used. A fresh detector tube was inserted into the pump inlet. Two minutes later, the concentration level at the interface where the stained reagent meets the unstained reagent was read. Before conducting experiments with plants, we have conducted a series of methodological experiments checking kinetics of O_2_ and CO_2_ concentration changes in sealed Petri dishes with no plants in them. After 2 days, the measured values were not different from those for respective atmospheric concentrations (20.9–21.0% and 420–425 ppm, respectively) and in a full agreement with reported literature data. This data was accepted as a “time zero” point. Four replicates were used for every treatment.

### Diaminobenzidine Staining

Diaminobenzidine (DAB) staining was performed as described previously (Chen et al., 2015). In brief, young seedlings were soaked in 1 mg/ml DAB solution (pH = 5.5) for 1 h and then immersed in 75% ethanol until the leaves turned white. The H_2_O_2_ signal (indicated by the pink or brown color) was observed under a stereoscope (Leica M205C). Eight seedlings were chosen for staining for every treatment and this experiment was repeated twice with similar results.

### RNA-Seq Analysis

To construct RNA libraries with a VAHTS mRNA-seq V3 Library Prep Kit for Illumina, 1 μg of total RNA was used. The procedure included polyA-selected RNA extraction, RNA fragmentation, random hexamer-primed reverse transcription, and 150 nt paired-end sequencing by Illumina HiSeq X-ten. Libraries were quantified using qPCR according to the qPCR Quantification Protocol Guide. To estimate expression levels, the RNA-seq reads were mapped to the *Arabidopsis* reference genome using TopHat, which is capable of reporting split-read alignments across splice junctions. Transcript counts were calculated, and the relative transcript abundances were measured in FPKM (Fragments Per Kilobase of exon per Million fragments mapped) using Cufflinks. We excluded transcripts with zeroed FPKM values of more than one for total samples. We added 1 to FPKM values of filtered transcripts to facilitate log2 transformation. Filtered data were transformed logarithmically and normalized using a quantile normalization method. For each transcript, we calculated fold change between case and control. Differentially expressed transcripts were determined by adjusting fold change ≥ 2 of more than at least one of total comparisons.

In total, eight steps were included: RNA extraction (Trizol method), rRNA and fragment removal, obtaining first-strand cDNA, obtaining second-strand cDNA, A-tailing addition, adapter ligation, purification and size selection and PCR amplification. Raw data were calculated as the FPKM of each transcript in each sample using Cufflinks software.

Gene ontology (GO) enrichment analysis was performed using the EasyGO gene ontology enrichment analysis tool^1^. The GO term enrichment was calculated using a hypergeometric distribution with a *P* value cut off of 0.01. *P* values obtained by Fisher’s exact test were adjusted with FDR for multiple comparisons to detect overrepresented GO terms (Xin and Zhen, 2007).

Three biological repeats were prepared for RNA-seq analysis and the data was generated in the same experiment.

## Results

### Effects of Different Sealing Methods on the Growth of *Arabidopsis thaliana*

**FIGURE 1:**
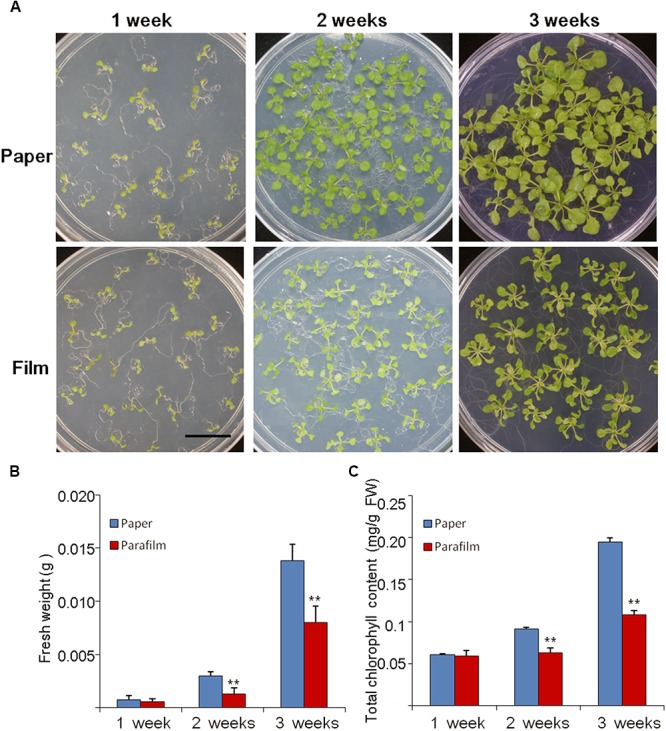
Phenotype **(A)**, biomass **(B)**, and chlorophyll content **(C)** of Col seedlings grown on MS medium for 1, 2, and 3 weeks with the different sealing methods. One (of six) representative plates are shown. For **(B)**, entire seedlings were used. Data are mean ± SD (*n* = 6). Data labeled with asterisks are significant at *p* < 0.05(Student’s *t*-test). For each treatment, seedlings in six plates were used for measurement and experiments repeated for three times with similar results.

The growth rate, fresh weight, chlorophyll content, and root length were similar between 7-day-old parafilm-sealed and paper tape-sealed seedlings. However, after 2 weeks of growth, the paper tape-sealed seedlings had more vigorous growth, higher biomass, and their shoots possessed more large green leaves (higher chlorophyll content, Figure 1C), compared with parafilm-sealed seedlings. The difference became even more prominent after 3 weeks of growth between seedlings using the two different sealing methods (Figure 1). Similar trends were observed for the root growth (Table 1). No difference in the root length/appearance was found between paper and parafilm-sealed seedlings after 7 days of growth, however, the root surface area, average diameter, root volume, and root tip number of paper-sealed *Arabidopsis thaliana* were greater than those of parafilm-sealed seedlings after 14 days of growth. After 21 days of growth, the root length, average diameter, root volume, and root tip numbers of paper-sealed seedlings were significantly greater than those of *Arabidopsis thaliana* with parafilm sealing (Table 1). These results indicated that plants grown with the use of the paper tape maintained a more advanced rooting system, and vigor as well as a higher proliferation capacity of *Arabidopsis* and the “excellent water vapor permeability” of parafilm may not be as good as the manufacturer mentioned in the description of the product.

**Table 1:** Effect of parafilm and paper sealing methods on parameters of the root systems of *Arabidopsis* seedlings after 1, 2, and 3 weeks in culture.

Treatment	Root length	Root surface	Average diameter	Root volume	Root tips number
Parafilm sealed-7 days	4.53 ± 0.349	0.20 ± 0.02	0.14 ± 0.001A	0.001 ± 0	44.51 ± 0.349
Paper sealed-7 days	4.52 ± 0.022	0.19 ± 0.004	0.13 ± 0.003B	0.001 ± 0	43.57 ± 0.022
Parafilm sealed-14 days	13.41 ± 0.373	0.548 ± 0.041B	0.126 ± 0.001B	0.002 ± 0B	56.75 ± 3.304B
Paper sealed-14 days	13.36 ± 0.217	0.74 ± 0.012A	0.14 ± 0.001A	0.003 ± 0A	71.33 ± 0.577A
Parafilm sealed-21 days	14.19 ± 0.64A	2.04 ± 0.187	0.31 ± 0.016B	0.01 ± 0.002b	114.67 ± 15.535a
Paper sealed-21 days	20.04 ± 0.751B	1.88 ± 0.016	0.43 ± 0.012A	0.02 ± 0.003a	148 ± 4.583b


*Root parameters include root length, surface area, diameter, as well as volume, and root tips number. Data is mean ± SD (*n* = 25 plants from three independent experiments)*.

*“a” and “b” indicates significant difference exists (*P* < 0.05), Student’s *t*-test; “A” and “B” indicates significant difference exists (*P* < 0.01), Student’s *t*-test*.

### Carbon Source Is an Important Factor That Influences Plant Growth

**FIGURE 2:**
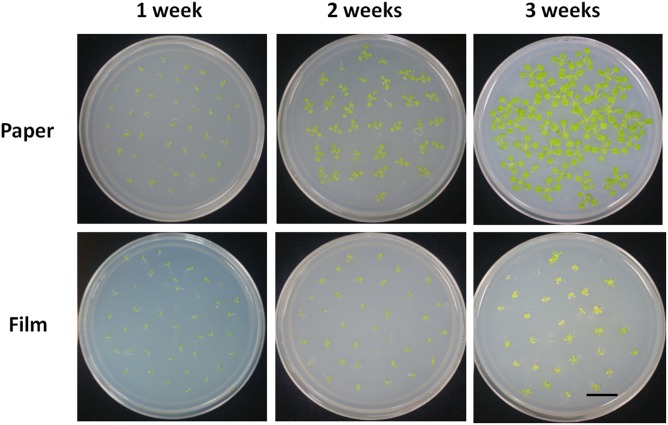
Phenotype of Col seedlings grown on MS medium without sucrose for 1, 2, and 3 weeks with the different sealing methods. For each treatment, four replicates were used.

Young seedlings grown in a Petri dish are frequently used for nutrition tests, because nutrient concentration in the media can be easily controlled. We tested the response of seedlings of the different sealing methods under sugar deficient conditions. We found seedlings in paper-sealed Petri dishes grew much better than those in parafilm-sealed ones after 2 weeks, the difference became even more pronounced after 3 weeks of growth (Figure 2). The most logical explanation for this fact would be a possible difference in the gas composition and its influence on the most important reactions in plants: photosynthesis and respiration. If this is the case, then these findings suggest that the starvation (carbon source cutdown) response may be closely linked with the gas environment. Possessing the impeded transpiration under sealed conditions, seedlings appear to have higher demands for nutrients and carbon. Therefore, false results may be obtained if this factor is not taken into account.

### Gas Content in Petri Dishes With Paper Tape and Parafilm Sealing

To test the above hypothesis that impeded gas exchange was a major factor differentiating between plant performance in parafilm- and paper-sealed Petri dishes, we measured the content of O_2,_ CO_2_, and ethylene in the Petri dishes with seedlings growing for 7, 14, and 21 days (Table 2). For O_2_ content, paper-sealed Petri dish consist more O_2_ compared to parafilm-sealed ones after 7-day growth. This trend reversed after 14 days growth and O_2_ concentration was the same at other time points. Ethylene cannot be detected during the whole growing process, despite the detection limit of the gas tube was as little as 0.05 ppm. CO_2_ content was significantly higher in parafilm sealed Petri dishes after 7-day (4.2% higher), and the difference became even higher at 21-day (33.9%) while it showed a trend reversal after 14 days growth (16.2% lower).

**Table 2:** Concentration of oxygen, carbon dioxide, ethylene in the Petri dishes, and moisture content in the media with seedlings growing for 7, 14, and 21 days.

Treatment	Parameter	7 days	14 days	21 days
Paper	O_2_ (%)	24.2 ± 0.1	20.7 ± 0.1	20.4 ± 0.2
Parafilm	O_2_ (%)	20.3 ± 0.1**	21.1 ± 0.1*	20.4 ± 0.2
Paper	CO_2_ (ppm)	528.1 ± 7	781.9 ± 19.8	410 ± 10
Parafilm	CO_2_ (ppm)	551.2 ± 7.5**	655.5 ± 34.5**	620 ± 20**
Paper	C_2_H_4_ (ppm)	Undetermined	Undetermined	Undetermined
Parafilm	C_2_H_4_ (ppm)	Undetermined	Undetermined	Undetermined
Paper	H_2_O (%)	96.2 ± 0.05	96.2 ± 0.48	96.1 ± 0.25
Parafilm	H_2_O (%)	96 ± 0.1	96.5 ± 0	96.3 ± 0.02
Paper	pH value	5.1 ± 0.1	4.8 ± 0.3	4.9 ± 0.2
Parafilm	pH value	5.4 ± 0.1*	5.2 ± 0	5.1 ± 0


*Mean ± SD (*n* = 6). Each experiment was repeated twice, with the same results*.

*^∗^*P* < 0.05 and ^∗∗^*P* < 0.01 indicate significant difference exists between different sealing method*.

The water content ranged from 96 to 97.2% and no difference was between different sealing methods. Msogoya et al. (2008) reported that autoclaving cause a significant drop of pH (from 6.8 to 5.8) value; different gelling agaents and autoclaving conditions resulted in different drop extent of pH value (Owen et al., 1991). In our experiment, pH value of media in parafilm sealed Petri dishes (7-day growth) was higher than paper tape sealed ones and no significant difference was found in other time points. Media pH level may influence nutrient uptake (Ramage and Williams, 2002), rooting and cellular growth (Leifert et al., 1992; Klerk et al., 2008), and plant gene expression (Lager et al., 2010).

### Effect of Different Sealing Methods on H_2_O_2_ Burst

**FIGURE 3:**
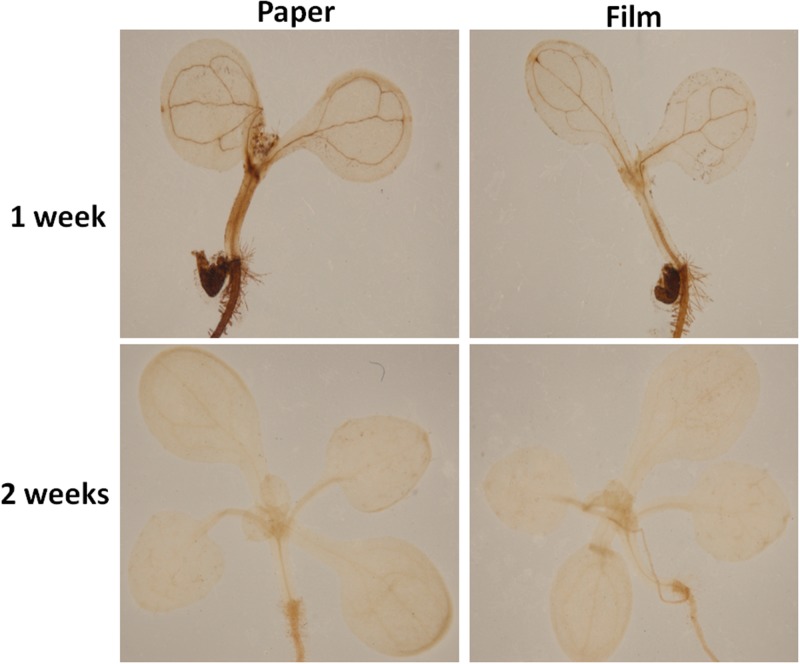
H_2_O_2_ accumulation in Col seedlings after 1 and 2 weeks of growth measured using DAB solution staining. DAB is oxidized by hydrogen peroxide in a reaction typically catalyzed by horseradish peroxidase (HRP). The oxidized DAB forms a brown precipitate, at the location of the HRP, which can be visualized using light microscopy. All seedlings were grown on standard MS media with sucrose shown in section “Materials and Methods”. One (of six) typical seedlings is shown for every treatment. The experiment was repeated twice with similar results.

Altered oxygen availability may potentially result in differential production of reactive oxygen species (ROS) by plant cells (Mehdy, 1994; Baker and Orlandi, 1995; Low and Merida, 2010). Thus, we tested H_2_O_2_ levels in parafilm and paper tape-sealed plants by DAB staining after 1 and 2 weeks of growth. As shown in Figure 3, after 7 days of growth, the production of H_2_O_2_, indicated by brown coloration in seedlings, was much higher in the leaf vain, central growth point and root of paper-sealed seedlings than that in those tissues of parafilm-sealed seedlings. In contrast, the H_2_O_2_ signal intensity was almost the same in 2-week-old seedlings using the two sealing methods (Figure 3).

Reactive oxygen species have also emerged as important regulators of plant development in which they play roles in processes as diverse as hormonal signal transduction and the modulation of cell wall polymer structure and tip growth (Potikha and Levine, 1999; Schopfer, 2001; Joo et al., 2005; Kerstin et al., 2009).

### Different Sealing Methods Influence Transcriptional Networks

**FIGURE 4:**
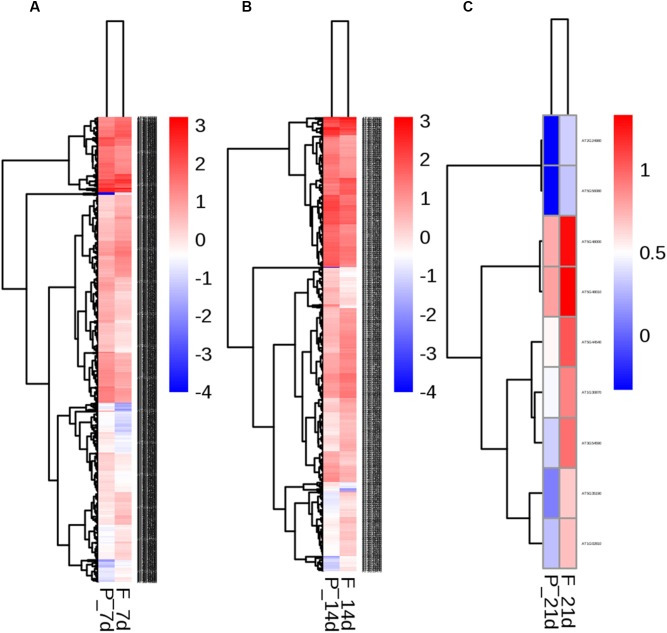
Two-way hierarchical clustering heat map of genes differentially expressed in seedlings in parafilm and paper tape-sealed petri dishes for 7 **(A)**, 14 **(B)**, and 21 days **(C)**. Differentially expressed transcripts were clustered. The color key in the right-hand corner is for the colors in the heat map. Detail information of these DEGs can be found in Tables 3–6.

**FIGURE 5:**
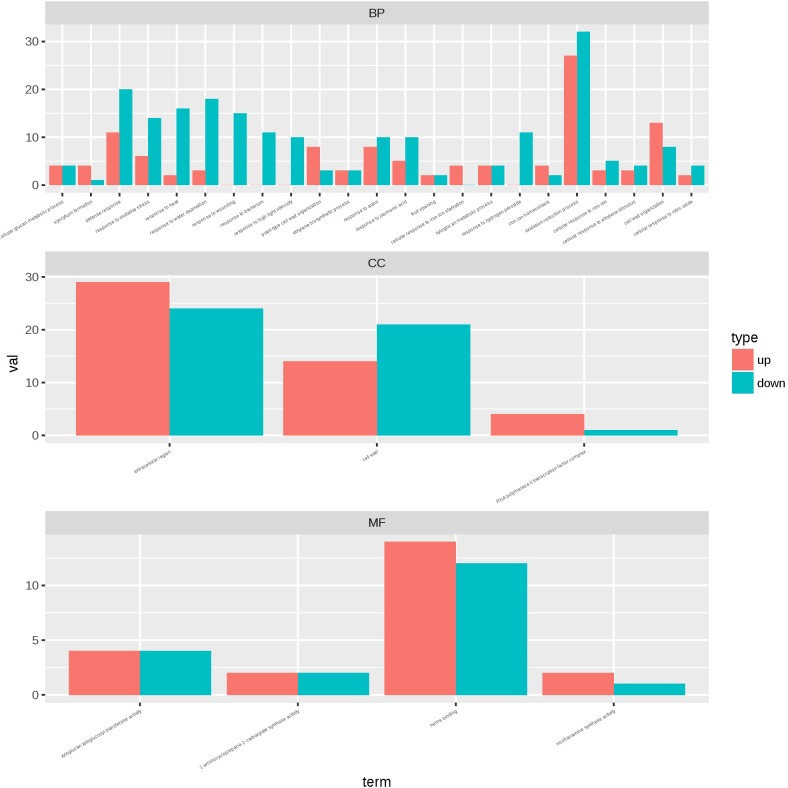
Gene ontology plant term enrichment. Genes differentially expressed in seedlings with the two sealing methods grown for 7 days were categorized and annotated based on biological processes, molecular functions, and cellular components. Three biological repeats were prepared for RNA-seq analysis and the data was generated in the same experiment.

To identify the transcriptional networks controlled during the growth process using the different sealing methods, we compared the transcriptomic profiles of whole seedlings of different age (1-, 2-, and 3-week-old) using genome-wide RNA-sequencing (RNA-seq) (Figure 1). RNA was isolated from a pooled seedlings sample of three biological replicates. We sequenced the libraries on an Illumina HiSeq 2000. The reads were aligned onto the *Arabidopsis* reference genome assembly (TAIR10), and the raw reads can be found in Supplementary Table S1. 725, 490, and 10 differentially expressed genes (DEG) were found in paper tape-sealed seedlings after 7, 14, and 21 days of growth, respectively, relative to parafilm-sealed plants. These genes were divided into two groups using a hierarchical clustering algorithm (Figure 4). Genes differentially expressed in seedlings with the two sealing methods grown for 7 days were categorized and annotated based on biological processes, molecular functions, and cellular components (Figure 5). The most impressive changes happened in defense, heat, and water response as well as oxidative stress response. Differentially expressed genes in seedlings grown for 14 days and 21 days with two sealing method categorized according to biological processes, molecular functions, and cellular components can be found in Supplementary Figures S1 and S2, respectively.

We listed all the DEGs in 7- and 14-day-old young seedlings (fold change > 2 or < -2), while little difference was found between 21-day-old plants using the different sealing methods. For paper-sealed 7-day-old seedlings, seven of cold-inducible genes, four of pathogenesis-related genes, three of heat shock genes, and several of stress-responsive genes (late embryogenesis genes and trichomes) and nutrition, phytohormone-responsive, lipid metabolism and development-related genes were downregulated compared to those in parafilm-sealed plants. In our previous study of the freezing tolerance of *Arabidopsis* sealed with parafilm in a Petri dish, we found it is very hard to obtain stable results using young seedlings. Therefore, we could not find the suitable treatment method to differentiate between wild-type plants and mutants, and we finished all the phenotypic experiments with adult plants in soil. From the current results, we may obtain some clue to explain why the phenotyping was so difficult, and the difficulty may result from the stress indicated by induce of stress responsive genes caused by the close culture system (Table 3). Similarly, after growth for 14 days, seven genes in the DREB family (involved in cold and drought tolerance), two pathogenesis-related genes, and several phytohormone-related genes (ET, JA, and cytokinin) were less expressed in paper tape-sealed plants compared to those in parafilm-sealed seedlings (Table 4). In contrast, in 7-day-old paper-sealed seedlings most upregulated genes were related to nutrient acquisition processes (iron deficiency responsive genes, phosphate responsive genes), defense-related genes, phytohormone-responsive genes and root development-related genes. The upregulation of BR increased expression of 1, GA synthesis (R2R3-MYB, also found in 14-day-old seedlings) and GA methyltransferase 2 (GAMT2, also found in 14-day-old seedlings) of paper tape-sealed plants and that might be a reason for better growth of seedlings (Tables 5, 6). Peroxidase 33, which is responsible for generating H_2_O_2_, was also much more elevated in paper-sealed than in parafilm-sealed plants and it may explain the increase in pink signal (Figure 3).

**Table 3:** Transcript profiles of key genes involved in selected biological processes (P_7d_vs_F_7d.DEG_down).

Category	Gene ID	Fold change	Annotation
Stress response	AT3G03341	-2.430404376	Cold-regulated protein
Stress response	AT2G42530	-2.380403804	Encodes COR15B, protects chloroplast membranes during freezing
Stress response	AT4G37220	-2.129808739	Cold acclimation protein WCOR413 family
Stress response	AT2G42540	-2.843307942	Cold-regulated 15a
Stress response	AT4G25470	-2.290807791	CBF2, Freezing tolerance QTL 4
Stress response	AT1G12610	-2.499310901	Member of the DREB subfamily
Stress response	AT1G33760	-3.649080447	Member of the DREB subfamily
Stress response	AT3G24500	-2.047880715	Multiprotein bridging factor 1C, elevated in pathogen infection
Stress response	AT1G66100	-3.840042699	Predicted pathogenesis-related protein. Belongs to thionin family
Stress response	AT5G36910	-2.026354501	Predicted pathogenesis-related protein. Belongs to thionin family
Stress response	AT2G26150	-2.940925461	Heat stress TF family. Response to misfolded protein accumulation
Stress response	AT5G52640	-2.154472808	Heat shock protein 90.1, required for RPS2-mediated resistance
Stress response	AT3G12580	-1.580271342	Heat shock protein 70
Stress response	AT5G51790	-1.719268011	Heat shock protein 70
Stress response	AT5G25240	-1.900316368	Stress induced protein
Stress response	AT1G01580	-3.231049455	Ferric reduction oxidase 2, ion transport, oxidation-reduction
Stress response	AT5G23990	-2.600346657	Ferric reduction oxidase 5, ion transport, oxidation-reduction
Stress response	AT5G05340	-2.006149033	Peroxidase 52, involved in lignin biosynthesis
Stress response	AT3G17520	-3.339407695	Late embryogenesis abundant protein (LEA) family protein
Stress response	AT1G52690	-3.449561191	Late embryogenesis abundant 7, LEA7
Stress response	AT1G12672	-3.512359022	Thionin-like protein
Stress response	AT5G44973	-5.991095858	Encodes a defensin-like (DEFL) family protein
Stress response	AT5G51440	-2.108071787	HSP20-like chaperones superfamily protein
Stress response	AT2G30432	-2.553689951	TRICHOMELESS1 D43, negatively regulates trichome formation
Stress response	AT5G24770	-3.499666348	Anti-insect activity. Induced by ABA, JA, salt, drought, wounding
Nutrition	AT4G31940	-8.311365602	Involved in the early Fe deficiency response
Nutrition	AT4G19690	-5.544586913	The gene encodes Fe^2+^ transporter protein
Lipid	AT5G62040	-2.975986193	PEBP (phosphatidylethanolamine-binding protein) family protein
Lipid	AT5G59310	-2.364963599	Bind fatty acids and acylCoA esters and transfer phospholipids
Hormone response	AT3G23230	-4.186210259	Member of the ERF subfamily B-3 of ERF/AP2 TF family
Hormone response	AT1G04180	-2.073351323	YUCCA 9, auxin biosynthetic process, oxidation-reduction process
Hormone response	AT3G63060	-2.048331651	EDL3 is an F-box protein involved that mediated ABA signaling
Hormone response	AT5G58310	-2.208068647	Methyl IAA esterase activity *in vitro*


*Genes differentially expressed in 7-day-old seedlings were functionally categorized according to gene ontology (GO) at the *Arabidopsis* Information Resource. The key components of selected biological processes are shown to estimate their biological relevance. Genes were chosen by fold change (FC) < -2 (paper tape-sealed seedlings compared to parafilm-sealed ones)*.

**Table 4:** Transcript profiles of key genes involved in selected biological processes (P_14d_vs_F_14d.DEG_down).

Category	Gene ID	Fold_change	Annotation
Nutrition	AT2G18700	-2.028886206	Enzyme putatively involved in trehalose biosynthesis
Stress response	AT1G66100	-3.994840224	Predicted pathogenesis-related protein. Belongs to the plant thionin (PR-13)
Stress response	AT3G62950	-3.532537012	Interact with the transcription factor TGA2 and suppress ORA59 promoter activity
Stress response	AT5G36910	-2.260143639	Predicted pathogenesis-related protein, belongs to the thionin (PR-13) family
Stress response	AT1G74930	-4.834086289	Member of the DREB subfamily A-5 of ERF/AP2 TF family
Stress response	AT5G06870	-2.283070222	Encodes a polygalacturonase inhibiting protein involved in plant defense response
Stress response	AT2G40000	-2.230629738	Defense response to bacterium, response to oxidative stress, SA, tryptophan
Stress response	AT1G74930	-4.834086289	Member of the DREB subfamily A-5 of ERF/AP2 TF family
Stress response	AT3G28740	-2.618735627	Member of the cytochrome p450 family, upregulated by *cis*-jasmonate treatment
Nutrition	AT3G57520	-2.086920131	α-galactosidase, breakdown raffinose into α-galatose and sucrose
Nutrition	AT4G13800	-2.102460777	Magnesium transporter NIPA
Phytohormone response	AT3G22275	-2.610242773	Jasmonate ZIM-domain (JAZ) protein 13, repressor of JA signaling
Phytohormone response	AT2G34600	-2.824291768	Jasmonate-ZIM-domain protein 7, JAZ7
Phytohormone response	AT1G75470	-5.252716971	Purine transporter, transport of purine and derivatives such as cytokinins
Development	AT2G32290	-2.128742351	β-amylase 6
Development	AT2G02710	-2.26492202	PAS/LOV protein, PLP, encodes a putative blue light receptor protein


*Genes differentially expressed in 14-day-old seedlings were functionally categorized according to gene ontology (GO) at the *Arabidopsis* Information Resource. The key components of selected biological processes are shown to estimate their biological relevance. Genes were chosen by fold change (FC) < -2 (paper tape-sealed seedlings compared to parafilm-sealed ones)*.

**Table 5:** Transcript profiles of key genes involved in selected biological processes (P_7d_vs_F_7d.DEG_up).

Category	Gene ID	Fold_change	Function
Stress response	AT2G41280	3.356066865	Hydrophilic protein similar to Late Embryogenesis Activated proteins
Stress response	AT5G44420	2.788950417	Low-molecular-weight cysteine rich 77, plant defensin 1.2
Stress response	AT1G13609	2.459469193	Defensin-like (DEFL) family protein
Stress response	AT5G67060	2.976341798	Defensin-like (DEFL) family protein
Stress response	AT3G55240	2.011985479	Overexpression leads to Pseudo-Etiolation in Light phenotype
Stress response	AT2G26010	3.380716537	Predicted pathogenesis-related protein. Belongs to PDF family
Stress response	AT5G63087	2.006385346	Plant thionin family protein
Stress response	AT3G49110	2.221092924	Peroxidase Perx33, generating H_2_O_2_ during defense response
Stress response	AT1G66390	3.084288122	ATMYB90, PAP2, production of anthocyanin pigment 2 protein
Nutrition	AT1G47395	2.388950877	Fe-uptake-inducing peptides 2, iron deficiency response
Nutrition	AT1G47400	2.803846696	Fe-uptake-inducing peptides 3, iron deficiency response
Nutrition	AT2G41240	2.070511343	Iron-deficiency key regulator
Phytohormone response	AT1G13430	2.541076433	Sulfotransferase, rise in response to cytokinin treatment
Phytohormone response	AT5G07310	2.340872366	Ethylene response factor 115
Phytohormone response	AT2G47870	3.378422467	ROXY5, interact with the TGA2 and suppress ORA59 promoter
Phytohormone response	AT1G68320	3.365487217	R2R3-MYB TF, phosphate starvation responses, and GA synthesis
Phytohormone response	AT5G56300	2.132370996	GAMT2, GA methyltransferase 2
Phytohormone response	AT2G22810	2.027242956	ACS4, key enzyme in the ethylene biosynthesis, induced by IAA
Phytohormone response	AT1G18400	2.224554893	BR enhanced expression 1
Development	AT4G30290	2.055149709	Xyloglucan endotransglucosylase/hydrolase
Development	AT1G17400	2.133846585	ATLAZY2, gravitropic response, and lateral root branch angle
Development	AT3G49970	3.213737238	Phototropic-responsive NPH3 protein
Development	AT3G50330	5.529546579	bHLH TF involved in transmitting tract and stigma development
Development	AT3G44990	2.157873748	Regulation of lateral root development and root growth pattern
Development	AT3G60650	2.33666671	Regulation of lateral root development and root growth pattern


*Genes differentially expressed in 7-day-old seedlings were functionally categorized according to gene ontology (GO) at the *Arabidopsis* Information Resource. The key components of selected biological processes are shown to estimate their biological relevance. Genes were chosen by fold change (FC) above 2 (paper tape-sealed seedlings compared to parafilm-sealed ones)*.

**Table 6:** Transcript profiles of key genes involved in selected biological processes (P_14d_vs_F_14d.DEG_up).

Category	Gene ID	Fold_change	Annotation
Stress response	AT1G14880	3.458062492	Plant cadmium resistance 1
Stress response	AT3G22231	2.136924387	Upregulated by virulent/avirulent Pseudomonas syringae pv. tomato
Stress response	AT3G55240	2.370781752	OE leads to Pseudo-Etiolation in Light phenotype
Stress response	AT5G42510	2.080222458	Disease resistance-responsive family protein
Stress response	AT5G05365	2.357149822	Heavy metal transport/detoxification protein
Stress response	AT4G33720	5.965728649	Pathogenesis-related 1 protein
Stress response	AT1G13607	2.477971817	Encodes a defensin-like (DEFL) family protein
Stress response	AT3G09922	2.353001629	ATIPS1, responsive to both phosphate and phosphite
Stress response	AT1G34510	2.493805491	Peroxidase superfamily protein
Stress response	AT1G66390	2.591469724	Production of anthocyanin pigment 2 protein (PAP2)
Nutrition	AT4G31940	4.308603234	CYP82C, involved in the early Fe deficiency response
Nutrition	AT1G24620	2.233682136	Root development under phosphate-deficient conditions
Nutrition	AT1G22150	2.088066424	Sulfate transporter 1
Nutrition	AT4G19680	2.63455365	Iron transporter, induced by iron and zinc deficiency
Nutrition	AT2G46860	2.53858144	Putative inorganic pyrophosphatase activity
Nutrition	AT3G49970	2.215488204	Phototropic-responsive NPH3 family protein
Nutrition	AT4G19690	3.141098196	Fe^2+^ transporter protein
Nutrition	AT1G61800	2.895154078	Glucose-6-phosphate/phosphate translocator 2
Phytohormone response	AT1G17710	2.618134662	Phosphoethanolamine/phosphocholine phosphatase
Phytohormone response	AT1G13430	2.741893253	Sulfotransferase, rise by cytokinin treatment
Phytohormone response	AT2G22810	2.054317395	ACS4, key enzyme in ET synthesis, induced by IAA
Phytohormone response	AT2G18010	2.5552925	Auxin-responsive protein family
Phytohormone response	AT2G41510	2.147965322	Cytokinin oxidase/dehydrogenase 1
Phytohormone response	AT2G41230	3.508303806	Regulator of sensitivity to ethylene and drought tolerance
Phytohormone response	AT1G13420	2.112264657	Sulfotransferase. Rise in response to cytokinin treatment
Phytohormone response	AT1G68320	2.20452222	R2R3-MYB TF. Phosphate starvation and GA synthesis
Phytohormone response	AT5G56300	2.02604329	GAMT2, Gibberrellic acid methyltransferase 2
Phytohormone response	AT5G21120	5.590716156	Ethylene-insensitive-like 2
Development	AT4G28850	2.842459027	Xyloglucan endotransglucosylase/hydrolase 26
Development	AT5G57530	2.673576546	Xyloglucan endotransglucosylase/hydrolase 12
Development	AT5G22410	2.166218317	Root hair specific 18
Development	AT4G02270	2.204978315	Seed and root protective protein, SRPP


*Genes differentially expressed in 14-day-old seedlings were functionally categorized according to gene ontology (GO) at the *Arabidopsis* Information Resource. The key components of selected biological processes are shown to estimate their biological relevance. Genes were chosen by fold change (FC) above 2 (paper tape-sealed seedlings compared to parafilm-sealed ones)*.

For the upregulated genes in 14-day-old paper-sealed seedlings compared to those in parafilm-sealed plants, most were involved in nutrient acquisition (iron transporter and phosphate-responsive genes), heavy metal transport genes (cadmium), and phytohormone-responsive genes (Table 5). After 3 weeks of growth, few differently expressed genes were found between plants of the two sealing methods, even though significant effects on growth were observed by naked eyes.

In summary, most abiotic stress-related genes were much more highly expressed in parafilm-sealed plants, while most nutrient responsive and growth promoting phytohormone genes were downregulated compared to those in paper-sealed seedlings. The expression patterns of these genes are consistent with the phenotype results.

## Discussion

Growth, development and quality of *in vitro* cultured plants are strongly influenced by their environment (Kristiansen et al., 1999; Mroginski et al., 1999). The chemical composition of the culture medium is generally accepted as of the utmost importance (Morard and Henry, 1998; Goleniowski and Trippi, 1999). External conditions, such as light, temperature and humidity, also have significant effects on plant growth.

In conventional tissue culture, the sealing film is usually used to seal the culture vessel, mainly to prevent pollution and excessive water evaporation, but a stressful environment is formed. Loose sealing can reduce the vitrification of carnations (Hakkaart and Versluijs, 1983; Dillen and Buysens, 1989) and promote the growth of seedlings. Lavender buds cultured in the “open culture system” with the appliance’s stomatal sealing have high vitality and regeneration rate (Tsuro et al., 2000), and our results demonstrated that parafilm might not be the optimal sealing method, because the seal is airtight and our RNA-seq results also demonstrated that fewer stress-related genes were highly expressed and that nutrition and phytohormone-responsive genes were upregulated in paper tape-sealed seedlings compared to parafilm-sealed plants after 1 and 2 weeks of growth.

More O_2_ content were detected in paper tape-sealed Petri dishes at early growth stage (7 days) and distinct CO_2_ content in parafilm-sealed and paper tape-sealed Petri dishes were measured. Since plants photosynthesize and need CO_2_ to generate glucose and store starch, and chemical reactions are pushed toward their end product, one would expect that more CO_2_ would be better, for increased growth and survival rates. However, it is not always the case, and it was shown that doubling the atmospheric CO_2_ concentration from 340–410 ppm to 610 ppm might have negative effect on plant growth and survival, and excess CO_2_ reduces the rate of transpiration of some plants (Mansfield and Majernik, 1970), and the water flow from the soil/medium to the leaves also drops, causing a runoff of water, and this in turn stalls nutrient uptake. In our case, the measured CO_2_ concentration in the parafilm-sealed Petri dishes reached 620 ppm level, so the above scenario may be plausible. It should be also noted that CO_2_ tolerances are species dependent. For example, CO_2_ tolerance in cotton plants is low, and starch buildup has been observed in the entire plant, but especially in the root systems and the stem (Hendrix et al., 1994). Future experiments and optimal and limiting CO_2_/O_2_ ratios for *Arabidopsis* plants, and link them with the changes in plant phenotype and gene expression changes reported in this work.

In addition, in order to observe root growth, the Petri dishes are oriented in a vertical position. Water easily accumulates at the bottom of a Petri dish and causes bacteria to multiply and serious stress on the root after long time culture. However, sealing with ventilated paper tape will keep the proper humidity of the medium, although the tape may fail for volatile substances in the medium, such as alcohol, and in antibiotic screening tests. However, we measured the moisture content of the medium sealed with two method, and we find the water content ranged from 96 to 97.2% and no difference was observed between different sealing method.

Inevitably, cultures enclosed in parafilm-sealed containers need a compensating pathway for diffusive gas exchange is contrived or replaced by some system of convective flow that carries gasses to and from the tissue. Obvious ways to achieve leakage include loosening the closure or inserting a gas-permeable membrane (e.g., polypropylene).

While parafilm-sealed Petri dishes are convenient and offer much advantages, they also possess a stressful environment and cause significant perturbation to plant gene expression profile. This alters plant metabolism and adaptive responses. Similar problems exist with the currently used Petri dish system with light inducing stress in roots. Simple darkenning of the root part is providing remedy. Qu et al. (2017) reported that an improved plant-growing method (IPG) based on a dark growth chamber found that the light intensity was dramatically reduced deeper in the dark chamber. The flavone, flavonol, and flavonoid biosynthesis pathways were significantly different between IPG and traditional method. Besides different gene expression patterns, compared to traditional plant-growing method (TPG), IPG produced plants with less total root length, lateral root length and root hair density, while their primary roots were longer. Moreover, root gravitropism, PIN2 (an auxin efflux carrier) abundance, H^+^ efflux or Ca^2+^ influx in root apices, were weaker in IPG-grown roots than those in TPG-grown roots (Xu et al., 2013). Yokawa et al. (2011, 2014) also reported that illumination of roots which naturally grow in darkness, even for a few seconds, induces an immediate and strong burst of ROS. In conclusion, in transparent Petri dishes, regular light exposure affects root morphology and behavior. An improved plant culture method using a shaded/darkened environment for roots that mimics normal underground light conditions was recommended (Yokawa et al., 2013). Besides, Buer et al. (2000) also reported that the gaseous environment within the Petri dish could be an important reason which causes the wave-like growth of *Arabidopsis thaliana* roots on semi-solid medium. Wrapping plates with surgical tape (loosely sealed) increased the wave characteristics and produced the slanting root growth wherein the wave response decreased in Nescofilm (airtight) sealed Petri dishes. Root response on tightly sealed plates could differ from responses on loosely wrapped plates. Plate wrapping materials, and other growth conditions occasionally, are taken for granted, while wide-ranging phenotypes require careful attention when results obtained from such systems are then extrapolated to glasshouse- or field-grown plants.

## Author Contributions

LX, SL, and WZ conceived and designed the research. LX and TJ conducted the experiments. WZ provided the reagents and items for the experiments. SL analyzed the data. LX wrote the manuscript. SS and WZ revised the manuscript. All authors read and approved the manuscript.

## Conflict of Interest Statement

The authors declare that the research was conducted in the absence of any commercial or financial relationships that could be construed as a potential conflict of interest.

## Footnotes

1 http://bioinformatics.cau.edu.cn/easygo/

## Supplementary Material

FIGURE S1 Gene ontology plant term enrichment. Genes differentially expressed in seedlings with two sealing methods grown for 14 days were categorized and annotated based on biological processes, molecular functions, and cellular components.

FIGURE S2 Gene ontology plant term enrichment. Genes differentially expressed in seedlings with two sealing methods grown for 21 days were categorized and annotated based on biological processes, molecular functions, and cellular components.

TABLE S1 RNA-seq reads.
