# Supplementary figures and images for: Plants Grown in Parafilm-Wrapped Petri Dishes Are Stressed and Possess Altered Gene Expression Profile
Source: Front Plant Sci. 2019 May 15;10:637. doi: 10.3389/fpls.2019.00637 (PMC6529517; doi:10.3389/fpls.2019.00637)

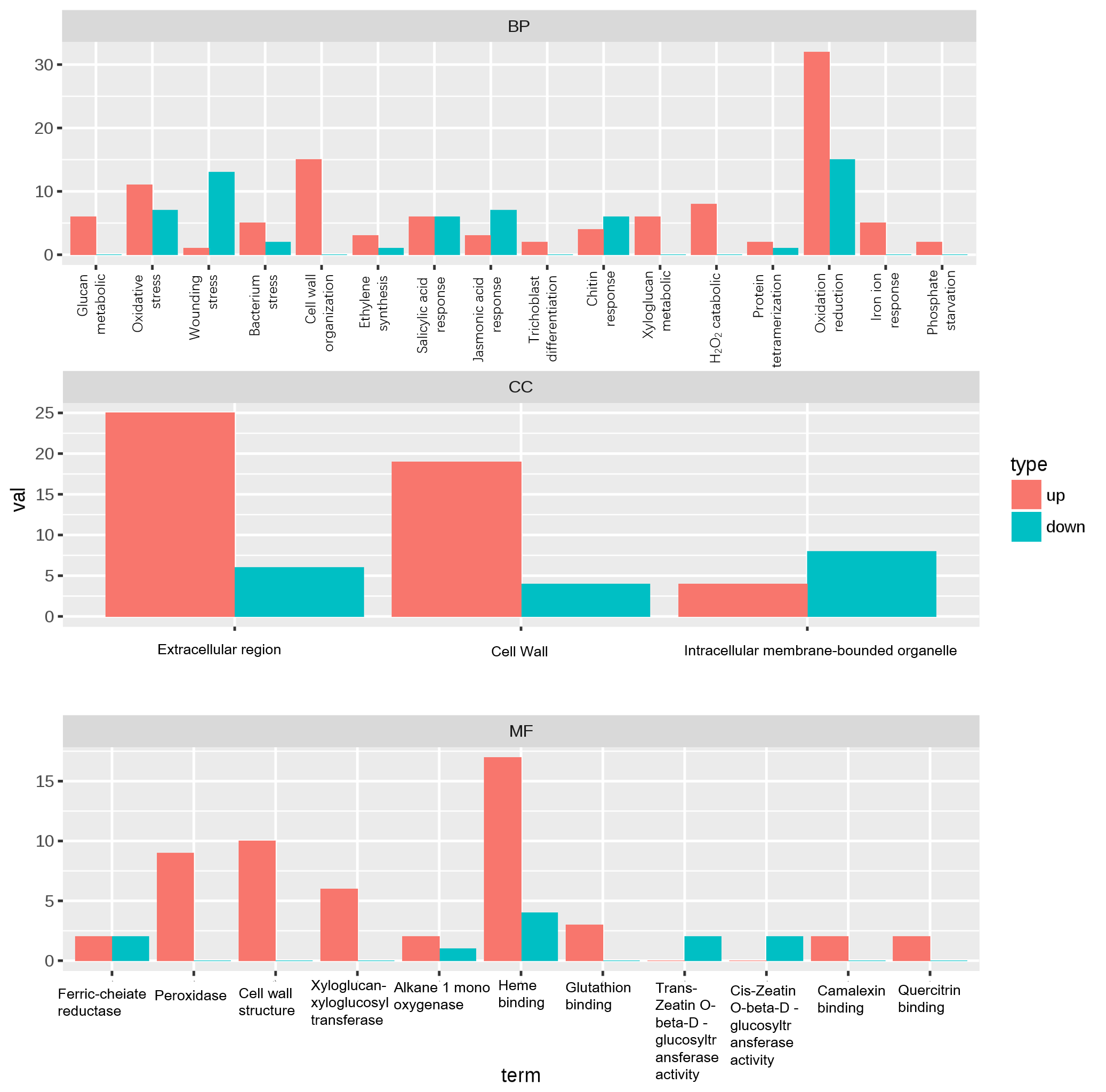

Supplement: FIGURE S1 — Gene ontology plant term enrichment. Genes differentially expressed in seedlings with two sealing methods grown for 14 days were categorized and annotated based on biological processes, molecular functions, and cellular components. [file Image_1.tif]

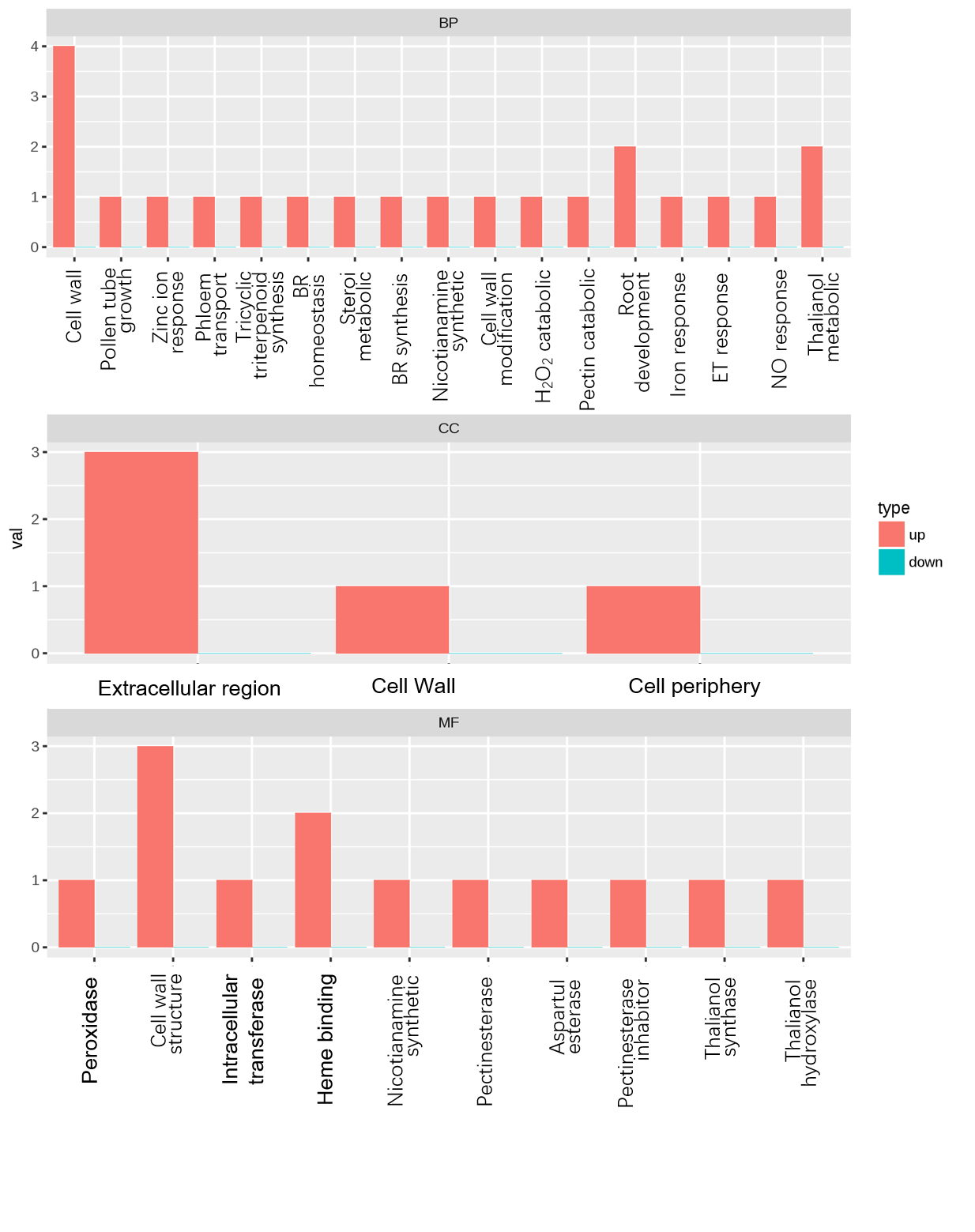

Supplement: FIGURE S2 — Gene ontology plant term enrichment. Genes differentially expressed in seedlings with two sealing methods grown for 21 days were categorized and annotated based on biological processes, molecular functions, and cellular components. [file Image_2.tif]
