# Supplementary material for: Plants Grown in Parafilm-Wrapped Petri Dishes Are Stressed and Possess Altered Gene Expression Profile
Source: Front Plant Sci. 2019 May 15;10:637. doi: 10.3389/fpls.2019.00637 (PMC6529517; doi:10.3389/fpls.2019.00637)
Supplement: TABLE S1 — RNA-seq reads. [file Table_1.DOCX]

**Supplementary Table 1. RNA-Seq reads**

| Sample name | Raw reads | Filtered reads | Reads with adapter | Reads with low quality | Reads with n rate exceed |
| --- | --- | --- | --- | --- | --- |
| P_7d_1 | 118442734 | 264836(100.00%) | 16376 (6.18%) | 40 (0.02%) | 248420 (93.80%) |
| P_7d_2 | 83085034 | 181226(100.00%) | 7922 (4.37%) | 32 (0.02%) | 173272(95.61%) |
| P_7d_3 | 87781562 | 194418(100.00%) | 10796 (5.55%) | 40 (0.02%) | 183582(94.43%) |
| F_7d_1 | 98831654 | 213690(100.00%) | 8864 (4.15%) | 34 (0.02%) | 204792 (95.84%) |
| F_7d_2 | 88782226 | 188806(100.00%) | 8872 (4.70%) | 48 (0.03%) | 179886(95.28%) |
| F_7d_3 | 87142642 | 188328(100.00%) | 8178 (4.34%) | 30 (0.02%) | 180120(95.64%) |
| P_14d_1 | 75984526 | 162482(100.00%) | 7912 (4.87%) | 32 (0.02%) | 154538(95.11%) |
| P_14d_2 | 76929858 | 165860(100.00%) | 7418 (4.47%) | 32 (0.02%) | 158410(95.51%) |
| P_14d_3 | 80185924 | 172676(100.00%) | 6968 (4.04%) | 34 (0.02%) | 165674(95.95%) |
| F_14d_1 | 70063792 | 154418(100.00%) | 7294 (4.72%) | 52 (0.03%) | 147072(95.24%) |
| F_14d_2 | 76270556 | 165410(100.00%) | 7164 (4.33%) | 42 (0.03%) | 158204(95.64%) |
| F_14d_3 | 80832328 | 170036(100.00%) | 6622 (3.89%) | 36 (0.02%) | 163378(96.08%) |
| P_21d_1 | 94873326 | 212560(100.00%) | 14716 (6.92%) | 36 (0.02%) | 197808 (93.06%) |
| P_21d_2 | 75384456 | 162312(100.00%) | 7182 (4.42%) | 24 (0.01%) | 155106(95.56%) |
| P_21d_3 | 77837482 | 169420(100.00%) | 8586 (5.07%) | 46 (0.03%) | 160788(94.90%) |
| F_21d_1 | 59859990 | 127656(100.00%) | 5324 (4.17%) | 36 (0.03%) | 122296(95.80%) |
| F_21d_2 | 76968938 | 164728(100.00%) | 5818 (3.53%) | 32 (0.02%) | 158878(96.45%) |
| F_21d_3 | 72615376 | 152296(100.00%) | 3478 (2.28%) | 16 (0.01%) | 148802(97.71%) |
